# PERFUMES: pipeline to extract RNA functional motifs and exposed structures

**DOI:** 10.1093/bioinformatics/btae056

**Published:** 2024-01-30

**Authors:** Arnaud Chol, Roman Sarrazin-Gendron, Éric Lécuyer, Mathieu Blanchette, Jérôme Waldispühl

**Affiliations:** School of Computer Science, McGill University, Montréal, QC H3A 0E9, Canada; School of Computer Science, McGill University, Montréal, QC H3A 0E9, Canada; Institut de Recherches Cliniques de Montréal (IRCM), Montréal, QC H2W 1R7, Canada; School of Computer Science, McGill University, Montréal, QC H3A 0E9, Canada; School of Computer Science, McGill University, Montréal, QC H3A 0E9, Canada

## Abstract

**Motivation:**

Up to 75% of the human genome encodes RNAs. The function of many non-coding RNAs relies on their ability to fold into 3D structures. Specifically, nucleotides inside secondary structure loops form non-canonical base pairs that help stabilize complex local 3D structures. These RNA 3D motifs can promote specific interactions with other molecules or serve as catalytic sites.

**Results:**

We introduce PERFUMES, a computational pipeline to identify 3D motifs that can be associated with observable features. Given a set of RNA sequences with associated binary experimental measurements, PERFUMES searches for RNA 3D motifs using BayesPairing2 and extracts those that are over-represented in the set of positive sequences. It also conducts a thermodynamics analysis of the structural context that can support the interpretation of the predictions. We illustrate PERFUMES’ usage on the SNRPA protein binding site, for which the tool retrieved both previously known binder motifs and new ones.

**Availability and implementation:**

PERFUMES is an open-source Python package (https://jwgitlab.cs.mcgill.ca/arnaud_chol/perfumes).

## 1 Introduction

Almost 75% of the human genome is believed to encode RNAs (Childs-Disney *et al* 2022), among which a majority is used to support non-coding functions. To perform these functions, non-coding RNAs rely not only on their sequence but also on their structure. Multiple layers of organization give each RNA a complex 3D shape that enables it to bind to proteins, other RNAs, or small molecules as a part of a biological pathway. First, canonical Watson-crick and Wobble base pairs quickly build a secondary structure, which is then used as a scaffold for building the 3D structure (Tinoco and Bustamante 1999). Non-canonical interactions (Leontis and Westhof 2001) often occur within loops of the secondary structure to stabilize their local 3D configurations and organize the global 3D architecture of the RNA. The conservation of the 3D structure of some of these loops suggests a possible role in the binding of the RNA with other molecules (Du *et al.* 2002, Huck *et al.* 2004, Thiel *et al.* 2018, Kalvari *et al.* 2020).

Several databases and tools have been developed to classify and predict the presence of these 3D motifs (Lemieux and Major 2006, Popenda *et al.* 2010, Chojnowski *et al.* 2013, Petrov *et al.* 2013, Ge *et al.* 2018, Reinharz *et al.* 2018). PERFUMES leverages those databases to unveil RNA 3D motifs that could be associated with a function. PERFUMES is a pipeline based on our previous 3D motifs prediction software BayesPairing2 (Sarrazin-Gendron *et al.* 2019, 2020) which can match motifs from a database to an input sequence using Bayesian networks as predictors. PERFUMES takes as input a set of sequences that have been associated with a given function of interest and identifies 3D motifs associated with this function. We illustrate the use of this software using an HTR-Selex experiment selecting for RNAs binding to SNRPA protein.

## 2 Materials and methods

PERFUMES takes as an input a (positive) set of RNA sequences associated with a biological function or property, and a set of background sequences used as a control. The output is a set of p-values representing the statistical significance (i.e. over-representation) of the occurrence of each motif in the positive dataset. It also provides metrics that help study the structural context of each significant motif.

**Figure 1.**
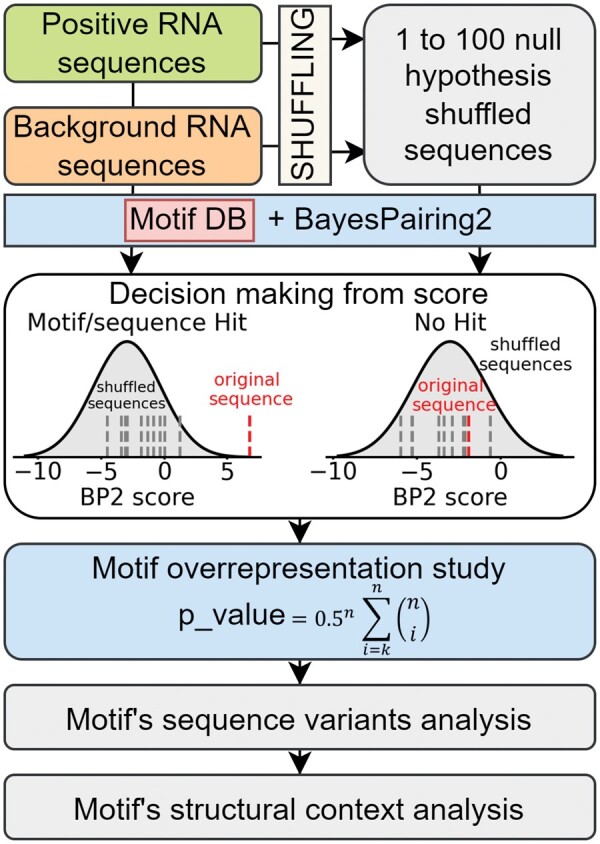
PERFUMES method pipeline.

The PERFUMES pipeline is illustrated in Fig. 1. The first step aims to determine a score threshold for each motif. Each input sequence is shuffled multiple times while preserving di-nucleotide frequency. Those shuffled sequences will serve as a control, as we expect the probability of occurrence of any arbitrary motif (predicted by BayesPairing2) to now be random. We run BayesPairing2 (Sarrazin-Gendron *et al.* 2019, 2020) on each shuffled sequence, and the scores of the motifs are modelled as a normal distribution. Then, we compute the score of the motif on the original sequence and compare it to the null hypothesis distribution. The latter gives us a *P*-value that can be used to filter the most significant predictions using a predetermined threshold.

The second step quantifies each motif’s enrichment in the positive sequences. For each motif, PERFUMES computes the number of positive and background input sequences containing a significant hit for the motif. The null hypothesis is that the presence of the motif in an RNA is unrelated to the property/function under study. This hypothesis is tested using a binomial proportion test; the associated *P*-value measures the significance of the motif’s enrichment.

The third step aims to characterize the sequence diversity at motif instances. For each enriched motif *m* (i.e. above our threshold) PERFUMES aligns the motif instances found in positive sequences to capture the sequence variations of those motifs. PERFUMES leverages this information to disentangle the selective pressure applied on the motif’s sequence only from the structural support encoded in the full sequence.

More specifically, given a motif, PERFUMES first retrieves all the sequence variants (of the motif) it identified in the two datasets and computes their enrichment factors in the positive dataset. This information helps determining if the sequence pattern alone can be associated with the sequence in the positive dataset.

Next, PERFUMES aims to determine if the folding of the complete sequence contributes to stabilize the presence of a motif at specific locations of the secondary structure. A first simple measure is to compute the energy of the minimum free energy (MFE) secondary structure compatible with the motif. Indeed, if a structured motif is functional, we hypothesize that the RNAs from the positive set should have a lower MFE than those coming from the background set.

Another more advanced metric evaluates the support from all structures present in the Boltzmann ensemble. PERFUMES computes the probability that the RNA folds into a secondary structure compatible with the motif as the sum of the probabilities of all of these structures in the Boltzmann ensemble. We obtain this value using the constrained folding option of RNAfold. Eventually, the user can choose different interpretations of compatible structures in which we require (or not) the secondary structure to include base pairs stacked on the motifs.

Lastly, for hairpin loops only, PERFUMES computes the average length of the helix surrounding the motif in compatible structures. This metric aims to provide a more interpretable measure of the structural support.

## 3 Results

### 3.1 Dataset

We illustrate the functionalities of our tool on a well-documented RNA motif that binds to the SNRPA protein, which is a component of the small nuclear ribonucleoprotein U1. SNRPA binds to an hairpin loop found as the stem loop II of the U1 RNA (Hall 1994).

We use a previously published dataset from an HTR-selex experiment that measured the binding affinity of RNA strands to the SNRPA protein (Jolma *et al.* 2020). The dataset can be found on European Nucleotide Archive (Accession: PRJEB25907). The authors used four cycles of successive amplification of the RNA, selection of the protein complexes, RNA extraction, and sequencing to assess the affinity of a pool of RNAs to SNRPA protein. Here, we use the sequences from the first cycle as the background set and those of the fourth cycle as the positive set. Because of the sheer number of sequences available, both the positive and negative sets were randomly downsampled to 5000 sequences.

As our motif dataset, we used BayesPairing2’s Reliable dataset, a dataset obtained from the 3D motif atlas. This processing removes motifs with insufficient numbers of unique occurrences (which would not allow statistically significant probability scoring of a candidate), removes motifs that are not compatible with the assumptions of BayesPairing (all motifs must have at least one unpaired base to be able to be searched by BayesPairing), and finally, splits motifs including occurrences from loops of different sizes into distinct motifs with the same underlying statistical model. This final dataset includes 271 motifs including hairpins and internal loops, covering a wide selection of the most represented structural motifs of the PDB database.

This dataset was chosen because it is the best PERFUMES-compatible, overall representation of the motifs found in known structures we have, and because these motifs specifically have been previously shown to be identifiable in structured RNAs, as this dataset corresponds to the benchmarked dataset in the BayesPairing2 publication (Sarrazin-Gendron *et al.* 2020).

### 3.2 Motif prediction

**Figure 2.**
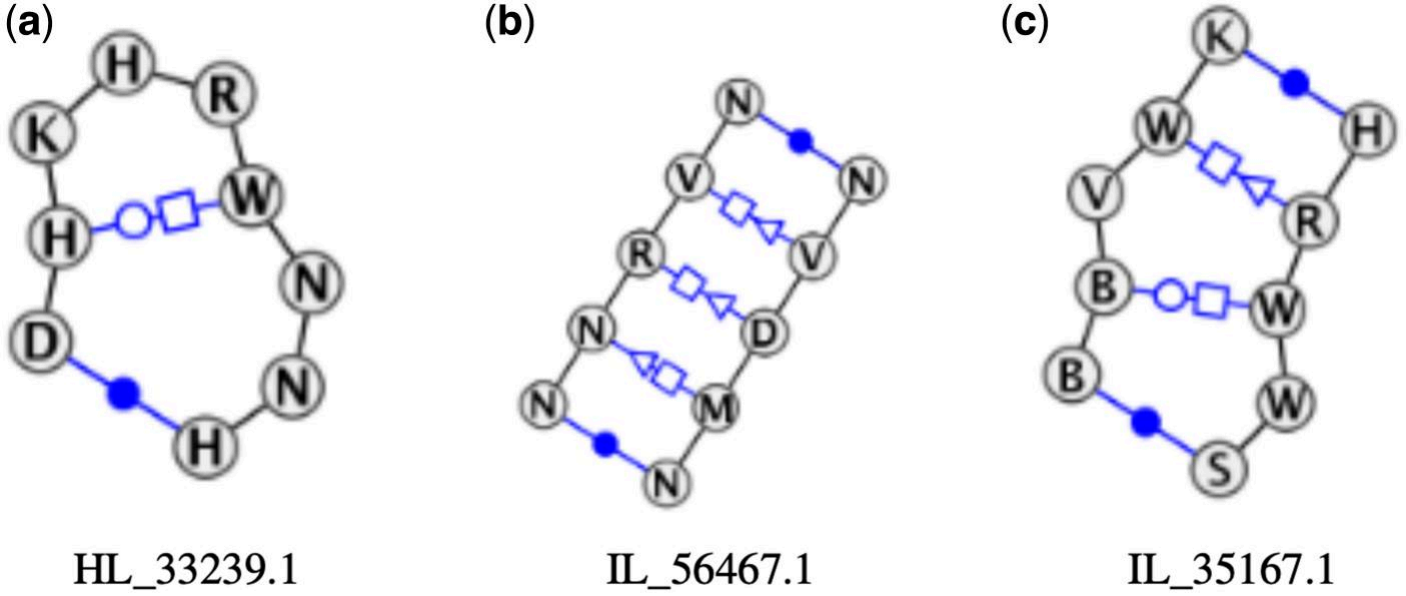
The three motifs identified by PERFUMES in the SNRPA binding HTX-Selex dataset. Images created with VARNA (Darty *et al.* 2009).

In our experiment, we use BayesPairing2 with a database of hairpins and internal loop motifs extracted from the RNA 3D Motif Atlas (Petrov *et al.* 2013) and set the threshold on the *P*-value for motif discovery at 0.05. PERFUMES identified three candidate motifs (Fig. 2): HL_33239.1 is a hairpin motif, while IL_56467.1 and IL_35167.1 are internal loop motifs. We then used PERFUMES’ post-processing functionalities to investigate these findings.

### 3.3 Analysis of hairpin motif HL_33239.1

HL_33239.1 is a T-loop with two stacked bulging bases. This motif contains a trans Watson-Crick/Hoogsteen non-canonical base pair between nucleotides 2 and 6. Nucleotides 7 and 8 are bulging out of the loop but stack together stabilizing the motif (Fig. 2a). The T-loop is known to be a stabilizing motif for the 3d structure (Chan *et al.* 2013).

**Figure 3.**
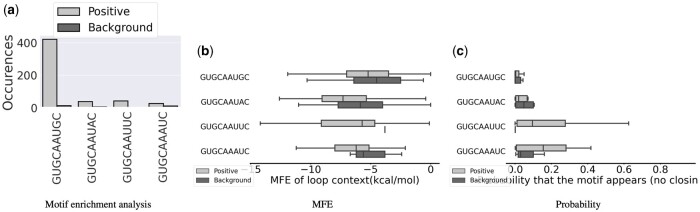
PERFUMES output for the HL_33239.1 hairpin motif. (a) Count of occurrence of sequence variants in each dataset. (b) Energy of the MFE structure compatible with the motif. (c) Probability of structure compatible with the motif.

Among the sequences in the positive set, PERFUMES identified several sequence variants of the hairpin motif. In addition to the sequence GCAC that has previously been associated with the binding to SNRPA (Hall 1994), PERFUMES also identified GCAA as a strong candidate (Note: as part of the larger RNA 3D motif Atlas motif). We calculated the enrichment of occurrences of the patterns GCAC and GCAA in the positive versus negative datasets to confirm this observation and found positive correlation supporting our findings (Fig. 3a).

We then look at the structural context of the four most frequently observed motifs. PERFUMES computes the energies of the MFE secondary structures compatible with the motifs in the positive and negative datasets (see Fig. 3b), and the probability of in the Boltzmann ensemble of all structures compatible with the motif (see Fig. 3c). In both cases, our results suggest that the structural context appears to help in stabilizing the results. The MFE of the secondary structures carrying the motif is consistently lower for sequences that bind SNRPA. The probabilities to fold in a secondary structure compatible with the motifs are more mitigated. The latter is significantly higher on two motifs but not on the two most frequently observed motifs. We hypothesize that these two hairpin motifs are more stable and require less structural support than the other.

### 3.4 Analysis of internal loop motifs

Now, we turn to the two over-represented 3x3 internal loops identified by PERFUMES in the RNAs datasets binding to SNRPA (i.e. IL_56467.1 and IL_35167.1). We show their structures in Fig. 2b and c.

**Figure 4.**
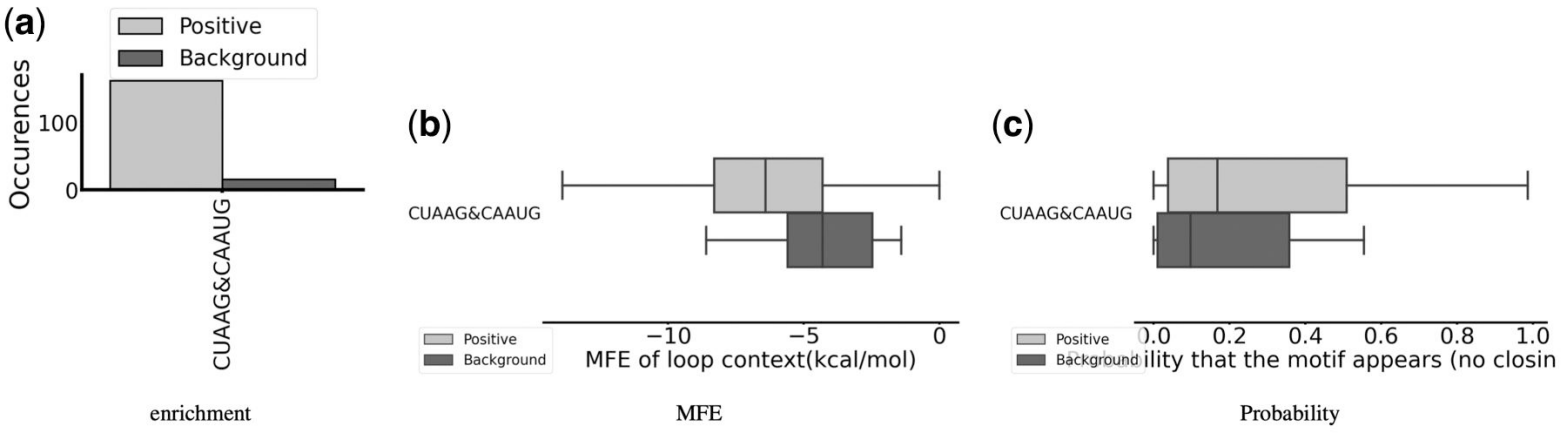
PERFUMES output for the IL_56467.1 3x3 internal loop motif. (a) Count of occurrence of sequence variants in each dataset. (b) Energy of the MFE structure compatible with the motif. (c) Probability of structure compatible with the motif.

Only one sequence variant of IL_56467.1 has been identified: CUAAG&CAAUG, and we show the results of the PERFUMES analysis in Fig. 4. The presence of this two-sequence motif is strongly enriched in the positive dataset (see Fig. 4a). Interestingly, we noticed that the second part of the internal loop has a similar sequence to those of the hairpin (i.e. CAAUG). As in the previous case, the MFE of the sequences carrying the motif in the correct secondary structure is lower than that of the sequences that bind to SNRPA, and the probability of secondary structures compatible with the motif is also higher. Here again, the significance of this motif thus appears supported at the sequence and structural levels.

**Figure 5.**
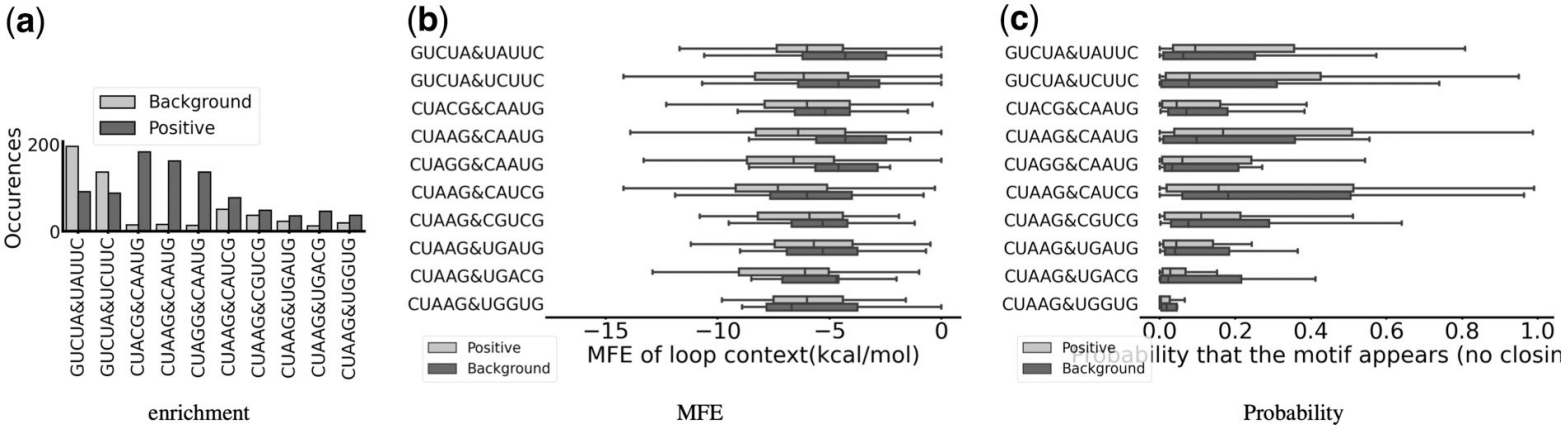
PERFUMES output for the IL_35167.1 3x3 internal loop motif. (a) Count of occurrence of sequence variants in each dataset. (b) Energy of the MFE structure compatible with the motif. (c) Probability of structure compatible with the motif.

The other motif, IL_35167.1, has many more sequence variants, which are analyzed in Fig. 5. This time some sequences are more frequently found on the negative set (i.e. background). Although nuanced, the structural analysis (MFE and probability of compatible structures) is more convincing, which highlights the benefits of our tool over pattern-matching tools.

We conducted a refined analysis of the relationship between the nucleotide composition of the motifs and the structural support (i.e. stability of compatible structures). Our results suggest that the occurrence of non-canonical base pairs might be associated with more stable structures (see Supplementary Material). Yet, these observations should be investigated further.

### 3.5 Comparison with other methods

We compared our results with the sequence motif discovery algorithm HOMER (Heinz *et al.* 2010). Unsurprisingly, HOMER identifies the same main sequences of the hairpin loop. In contrast, it could not retrieve the internal loop motifs found by PERFUMES. The details of this benchmark can be found in the Supplementary Material.

## 4 Implementation

PERFUMES is a python package that can be installed using pip. It comes with command lines to preprocess the dataset, runs BayesPairing2 on both the original and shuffled sequences, and finally analyses the results and automatically generates summaries as json and png files.

The execution of BayesPairing2 on all sequences can be parallelized to run independently on each sequence of the dataset. Our software uses SLURM, a job-queuing system used in cloud computing, but can be easily adapted to other environments (See GitLab documentation).

## Supplementary Material

btae056_Supplementary_Data

## Data Availability

The data underlying this article are available at https://jwgitlab.cs.mcgill.ca/arnaud_chol/perfumes
