# Supplementary material for: PERFUMES: pipeline to extract RNA functional motifs and exposed structures
Source: Bioinformatics. 2024 Jan 30;40(2):btae056. doi: 10.1093/bioinformatics/btae056 (PMC10868343; doi:10.1093/bioinformatics/btae056)
Supplement: btae056_Supplementary_Data [file btae056_supplementary_data.pdf]

PERFUMES: Pipeline to Extract Rna FUnctional Motifs and Exposed Structures

SUPPLEMENTARY MATERIALS

©ARNAUD CHOL, DECEMBER 11 2022

1 INTRODUCTION

Non coding RNA represent a large part of the RNAs in a human cell. Those RNA not only rely on their sequence to achieve a function but also on their structure. Indeed, multiple layers of organization give them a complex 3D shape that enables them to bind to proteins, other RNAs or small molecules as a part of a biological pathway. Starting with the secondary structure [Tinoco and Bustamante, 1999], the low bounding energy between A-U and C-G nucleotides, known as canonical Watson-crick base pairs will lead to well organised helices. Those helices are merged together by multiple loops composed of unbounded nucleotides. Those loops are call hairpins if they are closing a helix, internal loop if they connect two helices together, multi loop if they connect more than two helices, and finally bulges if they only have one free nucleotide that bulges out of an helix. In addition to this secondary structure, the free nucleotides of the loop are organized by non-canonical base pairs. Weaker, non canonical, bindings are constraining the nucleotides to adopt a specific conformation. Those loops and their set of non-canonical base pairs are believed to create specific 3D structures allowing for binding to other molecules. Some of those loops are shared between many species and are called RNA structural 3D motifs [[Thiel et al., 2018], [Kalvari et al., 2020], [Huck et al., 2004], [Du et al., 2002]]. Because being able to recognize and annotate those motifs would be a major asset for biology discovery, many databases are documenting those known and shared loops [[Chojnowski et al., 2013], [Lemieux and Major, 2006], [Ge et al., 2018], [Petrov et al., 2013a], [Popenda et al., 2010], [Reinharz et al., 2018]]. However, we still lack tools to enable a large scale use of those databases to find new functions associated with those loops. Our work tries to fill in this gap by leveraging those datasets to find over represented motifs in sets of RNA sequences. Such motifs can then be considered candidates for further studying before they can be fully annotated.

1.1 BayesPairing2

The pipeline we created is based around BayesPairing2's analysis of RNA sequences. BayesPairing2 [Sarrazin-Gendron et al., 2020] takes as input an RNA sequence and a database of RNA motifs and evaluates the likelyhood that each motif is present in the folding of the RNA molecule. The software uses Bayesian networks to model the possible mutations of a motifs that would not disturb its function. This notion makes use of base pairing isostericity [Westhof, 2014]. Combined with stochastic sampling of RNA secondary structures [McCaskill, 1990], BayesPairing2 outputs a matching score for a motif to a sequence.

1.2 Usage of this new tool

Our tool is a turnkey solution for anyone wanting to test the influence of a set of motifs in a biological function. Whether this function relates to protein binding, molecule lifetime, sub cellular location or any other the statistical method we developed holds. The input is a set of RNA sequences sharing a common function, and a set of background sequences, taken from the same context but not having the studied function. The output is a set of p-values representing the over representation of each motif. We also provide further analysis of the structural context of each significant motif, to really emphasise the difference with simple sequence motif search tools and ours. Those metrics are designed to prove the structural importance of the motif. They can also be used to design a new RNA sequence optimized for the motif to appear.

2 METHOD

2.1 Converting BP2 score into a boolean annotation

BayesPairing2 leverages both structural and sequence information to output its score. It first computes the partition function of the input sequence to then sample from the

Boltzmann distribution multiple secondary structures according to their probability. For each structure generated, it then finds all the internal and hairpin loops present in the structure and compares them to its database of motifs. Each loop is evaluated against all the motifs of the same size, plus or minus one nucleotide to account for a possible missing nucleotide or an additional one. The evaluation uses a Bayesian network to evaluate the probability that the motif of the database occurs given the sequence. Mutations from the original motif sequence generally lower the score but some mutations conserve the non canonical base pairing geometry and thus do not change the score significantly. This is why different sequences might be matched to the same motif. It then multiplies this probability with the probability that the loop was formed in the first place, given by the sampling algorithm.

The Bayesian networks BayesPairing2 uses to score a sequence are built by the software itself during installation. It builds each network from a set of sequences taken from a multiple sequence alignment corresponding to a conserved motif. The non-canonical base pair locations are known from the crystallography experiments, which allow to add edges to the network at those places. The conditionnal probabilities those edge encode can then be trained from the sequences themselves. Combining the precision of crystallography and a large number of sequences is only possible thanks to accurate alignment that ensures the non canonical base pairing will always take place at the same alignement indexes.

The first step of our tool is to convert the score BayesPairing2 outputs for each pair of input sequence and motif and convert it to a more statistically usable binary output. However, thresholding the score is not an option since its value depends heavily on the size of the motif, the number of non-canonical base pairs, the overall nucleotide content of the sequence and many other factors that are not directly related to the actual presence of the motif. BayesPairing2 is good to compare the presence of a given motif on multiple RNA sequences, or to compare the presence of multiple motifs on a single input sequence

but in our case both the motifs and the sequences are going to vary a lot, so the value of the score alone loses its value.

To combat this, each input sequence gets shuffled multiple times while conserving di-nucleotide content. Those shuffled sequences, although it has been shown they can adopt low energy structures [Rivas et al., 2017], have a negligible probability of having any motif present, because of the specific nucleotide sequence and the surrounding helical context needed to stabilize it. The distribution of output scores we observe on those shuffled sequences follows a Gaussian distribution, specific to each motif/original sequence pair. We consider this distribution to be the score distribution under the null hypothesis that the motif in not present in a sequence with the same di-nucleotide content as the original one. The score of the original sequence can then be computed. A p-value can be derived from the survival function of the Gaussian distribution we fitted to the shuffled examples. If the p-value is lower than a given threshold, then we consider the motif present in the original sequence.

Note that we derive a Gaussian null hypothesis score distribution of each pair of input sequence and motif. This model allows to sample only a few shuffled sequences in order to have a good estimate of the Gaussian parameters, reducing the computational costs. Sometimes BayesPairing2 does not output a score for a given motif sequence input, if no compatible loop was found to evaluate the Bayesian network on. We therefor include the probability that the motif is found by BayesPairing2 on the shuffled sequences and multiply the p value by this probability. Ending up in the following formula :

$$p_{value} = p_{found}.S_{\mathcal{N}_{(\mu,\sigma)}}(BP2(s)) \tag{1}$$

Where s is the input sequence, μ and σ the parameters of the normal distribution that models the negative score distribution, and S is the survival function.

Since we evaluate each motif against many sequences, we decided to correct the p values using a holm-sidak correction to update the p values according to the size of the input set of RNAs.

This score transformation greatly increases the computational cost, multiplying the number of sequences that have to be evaluated by BayesPairing2, but it gives more structural information to the results, by removing any side effect not due to the actual motif presence.

2.2 Statistical analysis of over representation

After BayesPairing has been run on all input sequences, we obtain a binary matrix of sequence/motif hits. For each motif m , we compute n_m the number of input sequences, coming from both the positive and the background set, carrying the motif, and k_m the number of sequences from the positive set carrying the motif. Our null hypothesis is that the presence of the motif in an RNA has no impact on the realisation of the function studied. In such case, the motif would be equally present between the positive set of RNAs and the background one. Under this null hypothesis, k_m is drawn from a binomial distribution of parameters $(n = n_m, p = 0.5)$.

We can test this hypothesis using a binomial test. The formula for our enrichment p_{value} is then

$$p^m_{value} = 0.5^{n_m} \sum_{i=k_m}^{n_m} \binom{n_m}{i} \quad (2)$$

Those p values once again need to be corrected to account for the multiple testing. We use a holm-sidak correction to update the p values at this stage.

This over representation metric is the first one computed by our tool after BayesPairing2 is finished running on all the sequences. It outputs a plot of the p_{values} of each motif and those above the threshold are considered candidate motifs for further analysis.

We observed unsurprisingly that most of the motifs have a high p value. Indeed, after the correction having a value lower than 5% is equivalent to having 13 matches in the positive set and only 1 in the negative one, or 66 to 34. Such over representations feel unrealistic if the motif has no effect on the function. This tool thus serves its purpose of narrowing down substantially the number of motifs that are worth investigating further.

2.3 Analysis of the structural context of the candidate motifs

At this stage, we have a list of candidate motifs that are over represented in the positive of RNA sequences. Since our goal is to find structural motifs, we wanted to have more metrics to understand how the structural context of the motif plays a role.

To be able to run more experiments on those motifs, we first need to retrieve the sequences on which they were found and the nucleotides where BayesPairing2 matched the motif.

For each motif m we collect the RNA sequences that had a p_{value} of matching to the motif lower than the threshold after correction. We run BayesPairing2 a second time, only on those untouched sequences. This time we collect the complete output of BayesPairing2, which includes the positions of the nucleotides the motifs were matched to. Sometimes as we mentioned earlier, BayesPairing2 will consider a nucleotide to be an addition from the original motif and will not include it in this table of matching nucleotides. We made sure to find such nucleotides and include them manually to the sub sequence of the RNA matching the motif. This way we have a set of sub-sequences found in our sequence dataset that were found to be the different variants of the motif in our dataset.

Our first analysis concerns sequence variants only, not the structure they adopt. For each motif we make a simple sequence search of all its variants in the whole dataset, and count how many times it was found in the positive set and the background one. This number is going to be higher than the number of hits BayesPairing2 gave, because it is only relying on sequence search. For internal loops, where the sequence variants are composed of two parts, separated by & symbol, we made sure that the first part of the

variant is found before the second part. We even count a minimum 4 nucleotide space between the end on the first part and the beginning of the second part to account for the maximum allowed bending of the RNA backbone.

This gives a measure of how each sequence variant is over represented in the positive set. A high over representation means that perhaps the structural context is not that important, and the presence of the sequence itself is enough to support the function. On the other hand if there is no over representation in the positive set, then it becomes more interesting. It means that having the sequence is not enough to support the function we study, but having the sequence in a structural context that makes BayesPairing2 match to a motif is the key to achieving the function. This is a good piece of evidence that the motif we are looking at is not only supporting the function because of its sequence, but also because of the structural context around it.

To further refine these results from BayesPairing2, which acts like a black box and does not give a lot of details about the exact structural context, we continue by computing 4 other metrics.

All of those four metrics involve computationally folding the RNA sequences using constraints. We ran a secondary structure prediction tool on the sequences and added constraints to force the motifs to appear where BayesPairing2 marked it in every fold generated. We can compute a few statistics on those folds and compare those statistics to those obtained when not having those constraints enabled.

We used ViennaRNA package [Lorenz et al., 2011] to compute those statistics. Using hard constraints, we start with a blank constraint '.' for all nucleotides, we then mark the closing basepairs of our motif with an '$<, >$' and the free nucleotides with an 'x'. We then take all the RNA sequences that we have found using the sequence only search and create a folding compound from those sequences with and without constraints.

First, we compute the minimum free energy (MFE) for the folding compounds with constraints. If the motif's structural context is important, we expect the RNAs from the positive set to have a lower MFE than the ones coming from the background set. Indeed,

RNAs from the positive set carry the function and therefor are supposed to have a great context for the motif, meaning that the MFE of the structure containing the motif is low.

However, this MFE metric is not perfect since it is not a local metric around the motif. The longer the RNA sequences are, the smaller the contribution of the motif in the MFE is. Also this metric does not look at the difference of MFE with and without constraints, but rather at the difference of MFE in motif compatible structures in the sequences that carry the function versus not.

As a second more comprehensive metric, we computed the exact probability that the motif appears in the folding of the RNA. That is the frequency of structures compatible with the motif in the Boltzmann ensemble. This metric involves computing the partition function of both folding compounds, with and without constraints, and the probability that the constraints are respected on an random fold of the molecule can be computed using this formula :

$$p = e^{-\frac{E_c - E_{nc}}{RT}} \tag{3}$$

Where E_c is the partition function of the constrained folding compound and E_{nc} the partition function of the unconstrained folding compound.

We expect this probability of presence to be higher for the RNAs of the positive set, under the hypothesis that they support the function because the sequence is designed to naturally have the motif in its correct structural context.

A slight variant of this metric we use is to only constrain the free nucleotides, but not the closing base pairs in the hard constraints given to ViennaRNA, this gives an insight of whether the exact structure of the motif needs to be present or if having the nucleotides exposed is enough to support the function.

Lastly, for hairpin loops only, we made a simple script that given an RNA sequence and constraints over the motif, samples secondary structures and compute the average length of the helix under the motif. We decided to allow for small bulges in the helix and simply counted the number of consecutive base pairs under the motif. The metric resembles the MFE metric but is much more local and gives details about the structural

context the motif is found. Once again, we except this helix to be longer in the RNAs coming from the positive set.

For all those metrics, we do not only consider sequences that BayesPairing2 has labelled carrying the motif, but any sequence carrying any sequence variant of the motif. This allows us to have sequences where the structural metrics would be bad and allow for a comparison with the better structural context metrics we expect to find in the positive set.

Table 1: Summary of the metrics computed by PERFUMES

Metric name	Description	Loop type
MFE	Minimum free energy of the structures compatible with the motif	Any
Probability of presence	Frequency of structures compatible with the motif in the Boltzmann ensemble	Any
Probability of free nucleotides	Frequency of structures compatible with the free nucleotides of the motifs, the ending base pairs may not be the same as in the motif	Any
Length of the supporting helix	Average number of stacked nucleotides under the motif's loop, we allow for single nucleotide bulges in the stack	Any

2.4 Implementation

Our tool is a python library that can be installed using pip. It comes with command lines to preprocess the dataset, run BayesPairing and the score transformation and finally to analyse the results and automatically generate results sumary in json files and plot all the figures in png format.

The long process of running BP2 for all sequences can be paralellized to run independently on each sequence of the dataset. Our software uses compute canada computing

power to parallelize this analysis. Each sequence analysis is a separate job, launched using the PERUFMES_ParseSeq command of the package. Each of those jobs can use multiple cpus to compute the scores for each of the shuffled sequences and the untouched sequence.

3 RESULTS

3.1 Validation on SNRPA binding data

Assembling the dataset

We decided to test our tool against a well documented RNA function : the binding to SNRPA protein. SNRPA is the protein part of the small nuclear ribonucleoprotein U1. Hall [Hall, 1994] has shown that it binds to an hairpin loop found as the stem loop II of the U1 RNA. This is the hairpin we are hoping to recover through our pipeline.

We used the data coming from the work of Jolma [Jolma et al., 2020] : an HTR-selex experiment measuring binding ability of some RNA strands to the SNRPA protein was conducted. Four cycles of successive amplification of the RNA, selection of the protein complexes, RNA extraction and sequencing were used to assert the affinity of an RNA pool to SNRPA protein. We used the sequences from the first cycle as the background set and those of the fourth cycle as the positive set. Although we also retrieved the cycle 0 sequences and re-ran the experiment with those and obtained similar results. However because of the number of sequences in this experiment, we had to down-sample to 5000 sequence only for each set, resulting in a total 10 000 sequences to be analysed. We used the first 5000 sequences of each file to simplify the matter, but one could rank them by sequencing quality for example.

To match the input format of our tool, we constructed a csv file containing an index, the sequence, a binary 1/0 notation for background/positive set. As the tool ask for

filters for dealing with more complex files, we just entered dumb filters stating that the set column must be lower than 2.

3.2 Hyper parameters used

Table 2: Hyperparameters used for SNRPA

Hyperparameter	Values
Bayespairing samplesize	5000
Motif database	RELIABLE
P value threshold	0.05
Number of input sequences	5000 positive 5000 background
Number of jobs	1000
cpu per job	8
Memory per job	8Go
Running time per job	30min

3.3 Detection of a well established binder motif

We ran our tool on compute canada. It took about 5 hours, mostly due to job allocation waiting time. The pipeline outputted three significant motifs for positive binding impact(fig 1). Their corresponding names in the RNA 3D Motif Atlas are : HL_33239.1, IL_56467.1 and IL_35167.1. 'HL' stand for hairpin loop and IL for internal loop, those are followed by an 5 digits identifier.

Because we were expecting to recover an hairpin loop, we first began by analysing HL_33239.1.

Sequence variants of HL_33239.1

HL_33239.1 is annotated on the RNA 3d motif atlas [Petrov et al., 2013b] as a T-loop with 2 stacked bulging bases. This motif contains a trans Watson-Crick/Hoogsteen non canonical base pairs between nucleotides 2 and 6. Nucleotides 7 and 8 are bulging out of the loop

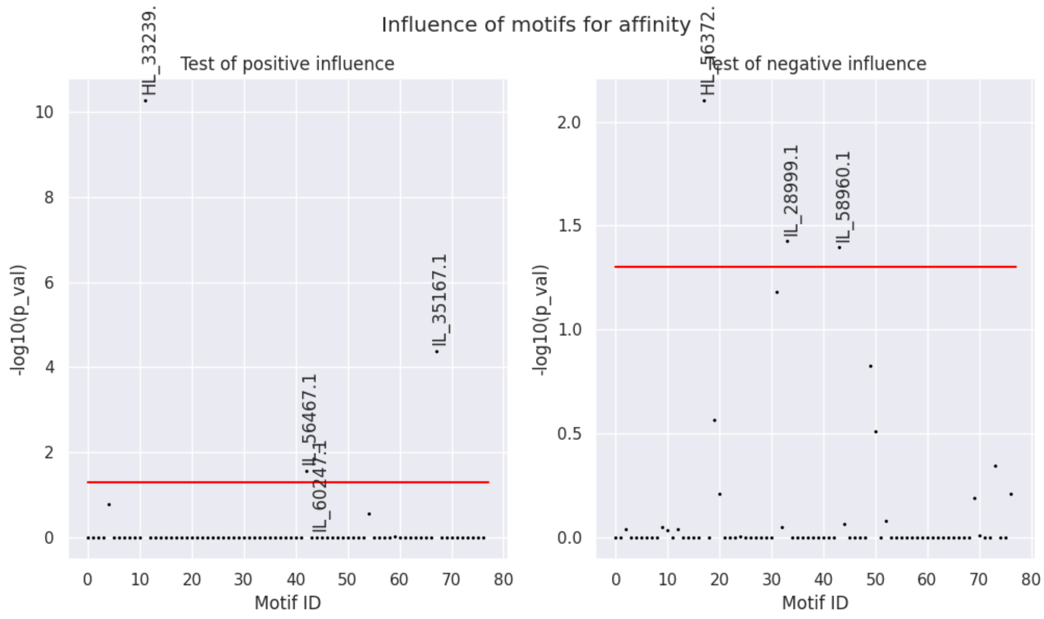

Figure 1: p values of the motifs for SNRPA.

but stack together stabilizing the motif (figure 2). The T-loop is known to be a stabilizing motif for the 3d structure [Chan et al., 2013].

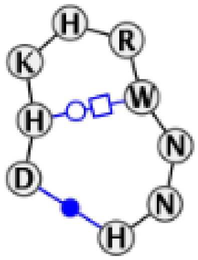

Figure 2: RNA 3D Atlas graph summary of HL_33239.1

First we ran BayesPairing2 on the sequences labeled as positive to the motif's presence, as discussed in the method section. Usually a motif has multiple sequence variants, this method, automated in PERFUMES, extracted the variants of our motif from the input set. The variants for this motif are shown in figure 3. Sequence GUGCAAUGC is the main variant in our set.

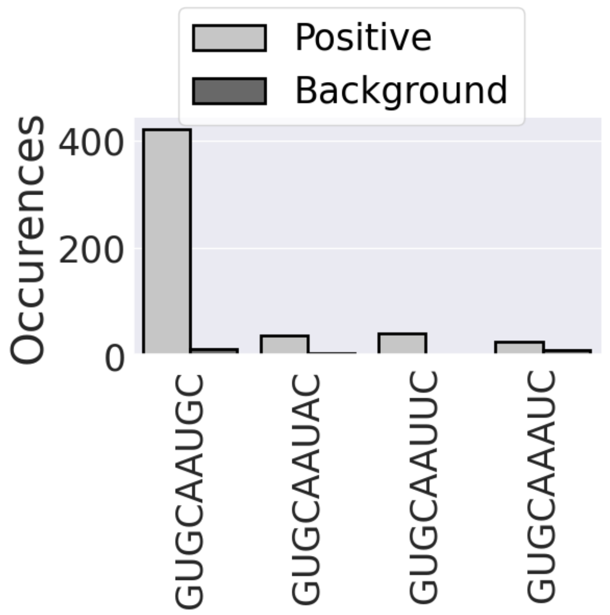

Figure 3: Sequence variants of HL_33239.1 in our dataset.

This surprised us a bit because we were expecting to find a GCAC sequence in the hairpin, to match the work of Hall [Hall, 1994]. We instead find GCAA. We wanted to double check this so we ran a simple script counting the occurrences of GCAC and GCAA (and some wider contexts as well) in both the full experiment files we downloaded (cycles 1 and 4) and obtained enrichment values shown in table 3.

Table 3: Sequence enrichement in the dataset

Sequence	# in positive	# in negative	Enrichment
GCAC	53908	62054	-14%
GCAA	192104	85894	x 2.23
UU**GCAC**	916	3622	-75%
GU**GCAA**	170184	16776	x 10.14
GGU**GCAA**UGC	1701	33	x 51.55

Those results support that our tool did find a good candidate, despite its difference with the documented motif. We ran the same experiment on a different HTR-selex experiment, specifically done for the PRIESSTESS [Laverty et al., 2022] paper, and obtained

similar results. Even worse, the larger motif UUGCAA, which is also labeled significant by PRIESSTESS is more common in the negative file than in the positive one. Note that the motif found by our method is 51 times more present in the positive dataset than in the negative one. This is over the complete dataset (not just 10000 samples subset) when using only the sequence and not even the structural context. At this point we decided to carry on with the structural analysis of the motif despite the difference with the literature as we obtained sufficient proof on the validity of this sequence.

Structural analysis of the motif

As discussed before the next step is to compare the structural contexts of those sequence variants when they are found on RNA sequences binding to SNRPA versus not.

Here is a quick summary of the metrics we developed to have a better understanding of why some RNAs in the negative set are not in the positive set despite having the sequence variants of the motif.

- *MFE when constraining the motif structure* : using the previously described constraints, if the MFE is higher in the RNAs from the negative set and lower in the positive set, it is an evidence that the nucleotides around the binder region have to be engineered so that they fold correctly and allow the binder to be exposed

- *Probability that the motif appears* : Using constraints we can compute the ensemble energy of the constrained foldings and all the other structures to compute a probability that the motifs will appear in the secondary structure. Once again we want this probability to be higher for good binders to prove that our motif need its structural context to be effective

- *Probability that the nucleotides of the motif are free* : Same test as before but we remove the constraints at the closing base pairs so that the motif is allowed to be larger

- *Length of the supporting helix* : When folding the RNA and constraining the motif to appear, we can compute the average length of the helix supporting the motif. This

is only implemented for hairpin. It gives a good local information about the way the motif appears in the RNA.

All of those statistics are shown in figure 4.

The sequence variants are represented from most present to less present in a top to bottom way. First, the length of the helix supporting the hairpin is longer for RNA sequences that bind to SNRPA for the two most present sequence variants. However this stack length is not high at all. Two possible explanations are possible: either there is usually an internal loop just after or the motif documented in our database does not reflect correctly the structural context of the actual motif that binds to SNRPA. We see here a first limitation to this metric, as we cannot distinguish between the two.

The MFE of the secondary structures carrying the motif is consistently lower for sequences that bind SNRPA. This is encouraging, and means that the nucleotides around the motifs are meant to form a low energy structure to stabilize our motif. Therefor we can argue that it is most plausible that the stack length is short because of an internal loop rather than a completely wrong structure.

However, when looking at the probability that the motif is present in the correct form, both the two less represented sequence variants have good results, but the two most represented ones do not. Indeed in either set, the probability that the motif appears is low. This means that even though from the previous plot we know that positive sequences are better at forming the motif from an energy point of view, in general the motif will not appear in the secondary structure. This questions the need for those sequence motif to be embedded in an hairpin to have a good binding affinity. A possible argument is that the folding on those RNA is first modified by an external product that could change the folding landscape and make the motif more stable. This can not be accounted by our current folding algorithm.

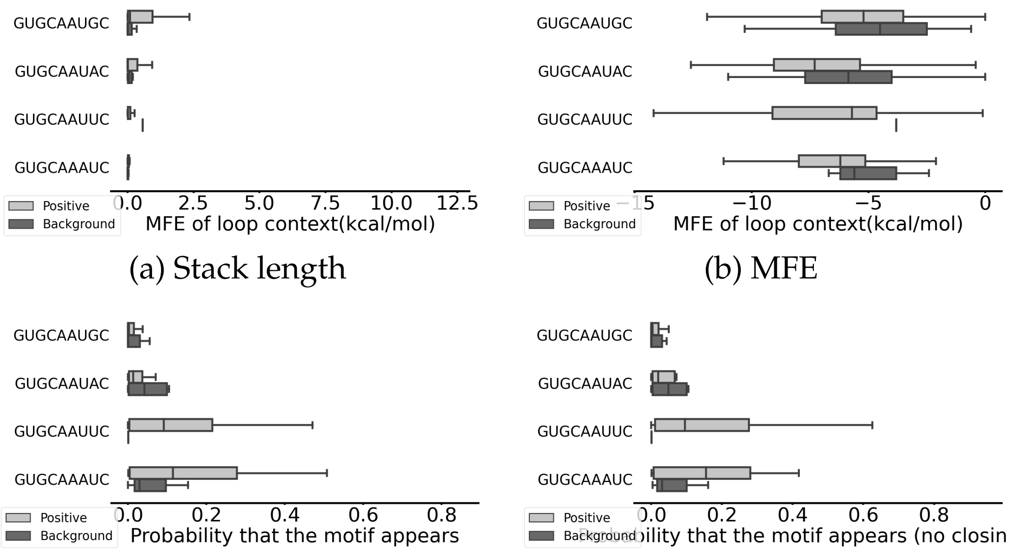

(a) Stack length (b) MFE

(c) Probability of presence (d) Probability of free nucleotides

Figure 4: Structural summary of HL_33239.1

3.4 Analysis of new binding motifs

Our tool also labelled two internal loops as significantly over represented in the RNAs binding to SNRPA. The two candidates are called IL_56467.1 and IL_35167.1.

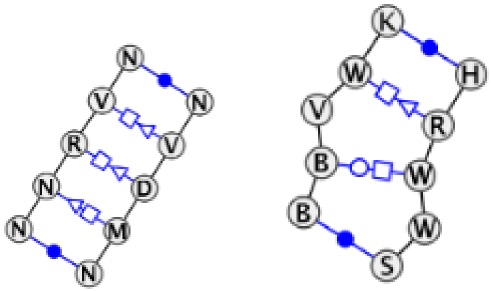

(a) IL_56467.1 (b) IL_35167.1

Figure 5: RNA 3D Atlas graph summary of the two new candidates

The first of them only has one sequence variant : CUAAG&CAAUG composed of CG closing base pairs with 3 free nucleotides on each side. The other one has multiple variants, one side closed by CG and the other sometimes closed by AU but mainly CG as

well.

The motif IL_56467.1 has a strong over representation sequence wise already, as shown in figure 6a. We noticed that the second part of the internal loop has a similar sequence to the hairpin : **CAAUG**. Similar to what we observed with the hairpin loop, the MFE of the sequences carrying the motif in the correct secondary structure is lower for the sequences that bind to SNRPA. This however does not mean that the motif is often present, indeed, the probability of presence is in fact lower for the sequences in the positive set than the background set. This show that even though the MFE of the secondary structures carrying the motif is still much higher than the global MFE of the sequences in general.

However, when looking at the probability that the nucleotides of the motif are free, we get a higher probability for sequences that bind SNRPA (figure 6d). Meaning that the sequences that bind SNRPA are constructed in a way that favors those nucleotides (**UAA** and **AAU**) to be free. Those results are still encouraging. We believe due to low number of motifs in BayesPairing2 database we used, we capture a significant motif that is close to one actually binding SNRPA but not exactly the same.

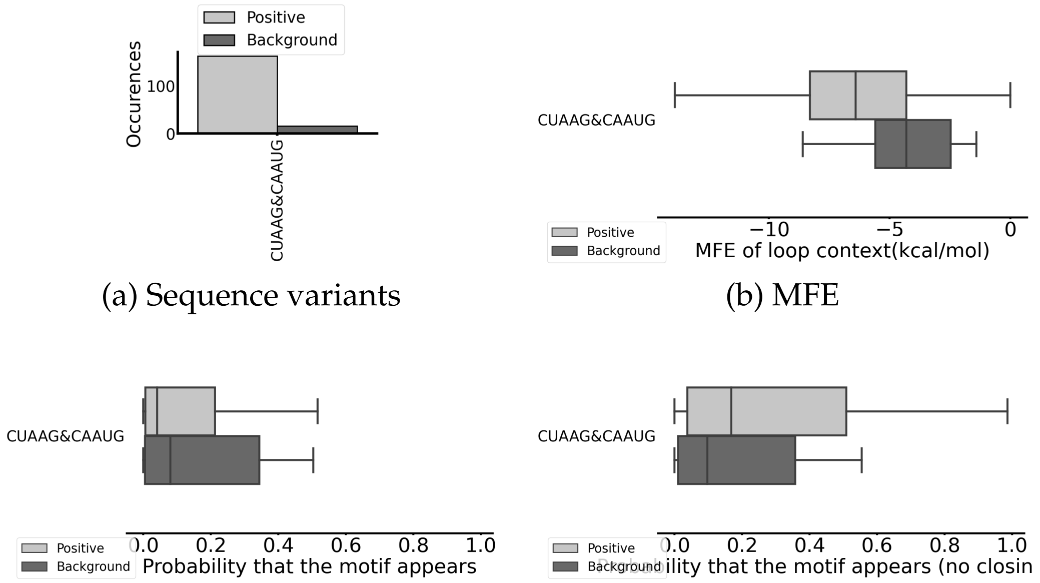

(a) Sequence variants (b) MFE

(c) Probability of presence (d) Probability of free nucleotides

Figure 6: Structural summary of IL_56467.1

The other motif, IL_35167.1 has more variants, but it is also an internal loop with 3 free nucleotides on each side. Some of the sequence variants are much more present on the negative set.
Therefor, we emphasise that sequence only search tools may not have been enough to pick up this motif. Take the most present motif, highly present in the background sequences, we will show in the next part that although **GUCUA** can be recovered using Homer, it is never seen combined with **UAUUC**. If the motif is proven to have a strong structural affinity then **UAUUC** might be as important as **GUCUA**.
Just like the two motifs before, this motif has lower MFE for sequences that bind to SNRPA. But this time, we also see that the probability that the motif is present is higher for sequences that bind SNRPA, especially for the most represented sequence variants. This result is better when looking at the probability that the nucleotides are free. Those differences of probabilities are not enough to be able to distinguish the sequences that will bind SNRPA from those who will not, which is the ultimate goal. They do show however some sign of structural properties that can lead to new experiment to better test the affinity of SNRPA to the sequence and to the structural part of the motif separately.

3.5 Interpretation regarding non canonical base pairs energy

Since the structural context experiment did not turned out as we expected, we decided to compare the frequency of compatible structures in the positive set of RNAs versus shuffled versions of those same RNAs. This is different from the previous experiment where we compared them to RNAs that do not bind to SNRPA. In those shuffled versions, we would shuffle the nucleotides around the motif but leave the motif intact. We wanted to see if the nucleotides around the motifs were increasing the frequency of a compatible structure from a random context.

The results are presented in figure 8. We also highlighted the motif's non canonical base pairings we found on the RNA 3D Atlas database.

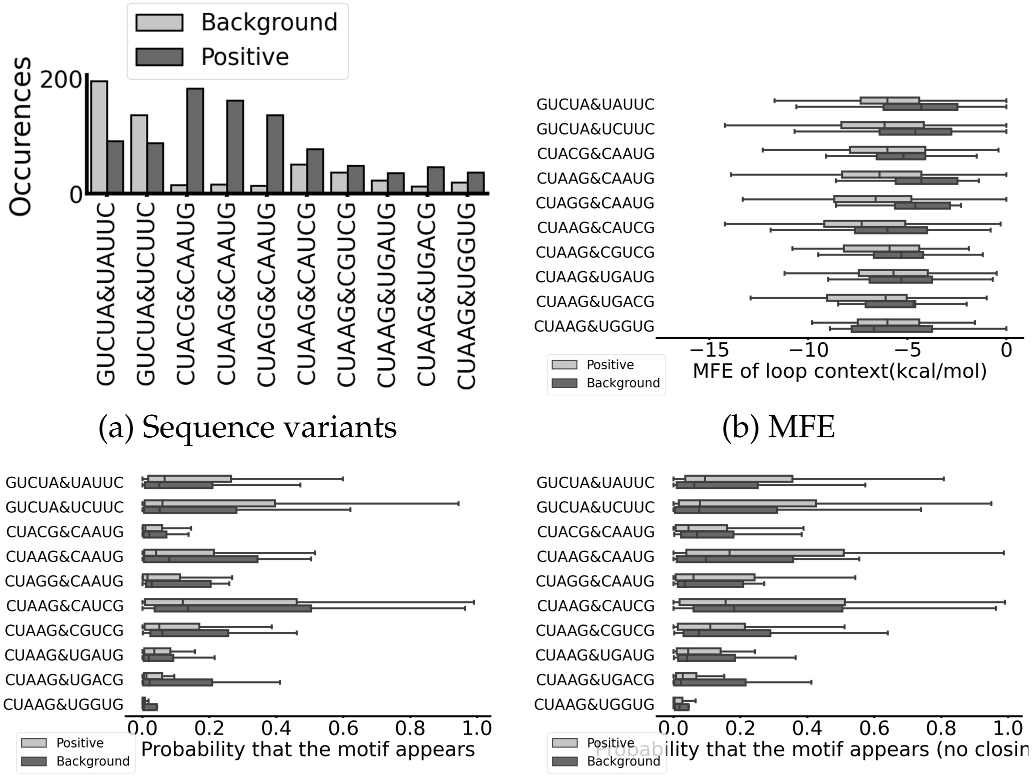

(a) Sequence variants (b) MFE

(c) Probability of presence (d) Probability of free nucleotides

Figure 7: Structural summary of IL_35167.1

While we expected the difference between the natural and shuffled sets to be higher, it was not really the case for a number of variants. For many of the variants, the frequency of structures compatible with the motif is comparable with or without shuffling the nearby nucleotides. This would mean that the context is not build to support the motif. Only we can notice some variants were it is not the case and we have a higher probability of compatible structures for the natural sequences, for example CUAAG&CAGUG for IL_35167.1.

This internal loop is a stack of non-canonical pairing. On the variant with the highest difference between shuffle and natural context, the non canonical base pairs are UG for trans Watson Hoogsteen and AA for trans Hoogsteen Sugar. AA for tHS is way less common than than AG (see [Berman et al., 1992]), and thus probably of higher energy, this would mean that to maintain the motif, the nearby nucleotides would have to take over

to stabilize the motif, and this additional stability would this time be considered by the folding model. Same thing with the tWH base pair, UA is the most common conformation, the lowest energy one, so if it is instead UG, less common and thus of higher energy, the nearby nucleotides would have to compensate.

We observe that when neither non canonical base pairs are in the lower energy conformation, our model is much more capable of differentiating natural RNA sequences than when they both are in the most common conformation. Our folding model ignores those non-canonical base pairs, and when those non canonical base pair do not play a significant role in lowering the energy, our model indeed find a supporting structural context around the motif. We think that the folding model not having the energy parameters of our motifs is what currently prevent us from having better metrics of the structural context.

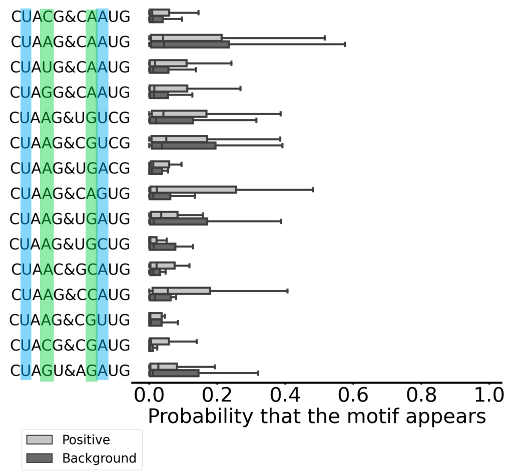

(a) IL_35167.1

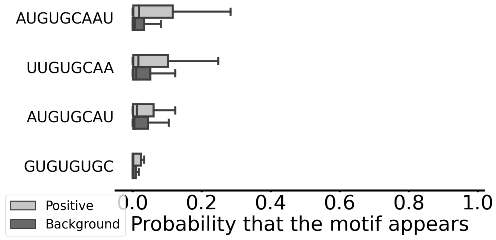

(b) HL_08510.1

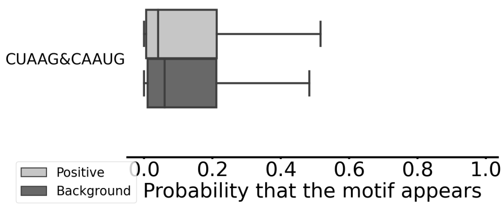

(c) IL_56467.1

Figure 8: Frequency of compatible structures in Boltzmann ensemble for the positive RNAs versus shuffled contexts versions of those same RNA sequences. Theoretical motif's non canonical stacking is highlighted when applicable.

3.6 Comparison to existing methods

Homer [Heinz et al., 2010] is a tool that leverages sequence only information to find over-represented motifs in a sequence set versus the other. The motifs for Homer are position weight matrices that can be used to scan a sequence and get the matches. We used this tool to compare our results. We find that the main sequence of the hairpin loop we found using our method is also found be homer, confirming our previous results. Also some sequences of the hairpin loops are also similar, like GUCUA of motif 5 or the CUAAG of motif 6. While those motifs can certainly expand further than our that are constrained to stop at the base pairs, they miss the complete sequences of the internal loops that have two separate parts. Motif 6 for example could have much more over representation (somewhere like 4% vs 0.2% instead of 1.9% to 0.43%) if it was able to cover the CAAUG (the other part of the hairpin loop) instead of U——CUUA the nucleotides that surround it.

PRIESTESS is a motif searching software that incorporates structure information into classical sequence motif search tools but using a more complex alphabet to describe the nucleotides. We wanted to run it on the dataset we assembled from the same experiment they used for their paper but unfortunately we got an error making it impossible to run our self. This would have enabled us to double check what is happening with the GCAC instead of GCAA situation.

4 CONCLUSION

Our tool is a ready to use python script that should enable people to rapidly evaluate the possible implication of RNA motifs in the biological function they are monitoring. Its ease of installation and use will hopefully allow more people to plug their database and discover new roles for previously known motifs. We also think this tool could be linked to some motif annotation database to allow for a first insight in the biological pathway underlying the studied function.

Rank	Motif	P-value	$logP_{value}$	Targets	Background
1	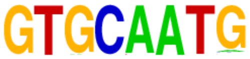	1e-561	-1.293e+03	52.24%	5.08%
2	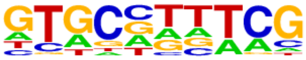	1e-24	-5.653e+01	2.42%	0.13%
3	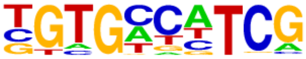	1e-18	-4.357e+01	6.60%	2.58%
4	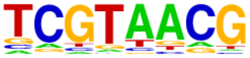	1e-12	-2.883e+01	8.34%	4.53%
5	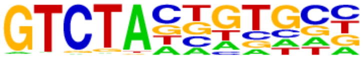	1e-12	-2.774e+01	1.86%	0.34%
6 *	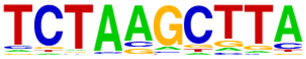	1e-10	-2.380e+01	1.90%	0.43%
7 *	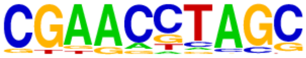	1e-9	-2.136e+01	1.16%	0.14%
8 *	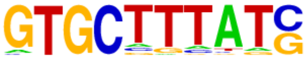	1e-9	-2.097e+01	0.84%	0.05%
9 *	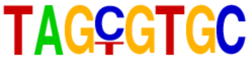	1e-8	-2.067e+01	1.80%	0.45%
10 *	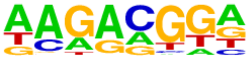	1e-7	-1.634e+01	1.94%	0.67%
11 *	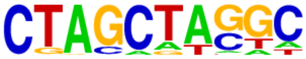	1e-6	-1.474e+01	1.00%	0.20%

On our test data, we have been able to recover known motifs that bind to SNRPA. In addition to the sequence information, we also have access to a lot of insight to the structural context those motifs are embedded in. Those metric can be used to confirm the structural importance of some binder motifs, and be used as a starting base to design even better binders, by optimizing a sequence to have the motif embedded in the most favorable context.

Unfortunately, our structural metrics did not show as clear of a distinction between the favorable and unfavorable contexts. However, by comparing the contexts of the binder motifs to shuffled contexts, we noted some interesting correlation between a bad performance and the presence of non-canonical base pairs our energy model does not account for.

As we said in the introduction, those motifs help stabilizing RNA molecules by providing additional alternative stacking. Thus our 2D structure prediction is off because it does not know the energy parameters of such structures. It would be interesting to compare the effect of a motif stabilized by external stacking versus stabilized by a weaker stacking compensated by a good non canonical pairing, to see if the function remain as strong.

We hope this project can be revisited with different target and maybe different energy models to be able to come up with a better model of the motif structural context.

5 ACKNOWLEDGEMENTS

I would like to thank my supervisors Prof. Jerome Waldispühl and Prof. Mathieu Blanchette for all their help and advice with this Master. I would also like to thank other people from the lab and its collaborators who have contributed to answering any question I had, those include Prof. Vladimir Reinharz, Roman Sarrazin-Gendron, Dr. Hua-Ting Yao and Prof. Yann Ponty. I also appreciate all the support I received from the people I worked along side to : Etienne Reboul and Paul Andre Henegar.

This research was enabled in part by support provided by Calcul Quebec (calculquebec.ca) and Compute Canada (computecanada.ca).

Conflict of interest statement.

None declared.

Data availability.

https://jwgitlab.cs.mcgill.ca/arnaud_chol/perfumes

Bibliography

H.M. Berman, W.K. Olson, D.L. Beveridge, J. Westbrook, A. Gelbin, T. Demeny, S.H. Hsieh, A.R. Srinivasan, and B. Schneider. The nucleic acid database. a comprehensive relational database of three-dimensional structures of nucleic acids. *Biophysical Journal*, 63(3):751–759, 1992. doi: https://doi.org/10.1016/S0006-3495(92)81649-1. URL `https://www.sciencedirect.com/science/article/pii/S0006349592816491`.

Clarence W Chan, Bhaskar Chetnani, and Alfonso Mondragón. Structure and function of the t-loop structural motif in noncoding rnas. *Wiley Interdisciplinary Reviews: RNA*, 4(5): 507–522, 2013.

Grzegorz Chojnowski, Tomasz Waleń, and Janusz Bujnicki. Rna bricks - a database of rna 3d motifs and their interactions. *Nucleic acids research*, 42, 11 2013. doi: 10.1093/nar/gkt1084.

Zhihua Du, Kenneth E Lind, and Thomas L James. Structure of tar rna complexed with a tat-tar interaction nanomolar inhibitor that was identified by computational screening. *Chemistry & biology*, 9(6):707–712, June 2002. doi: 10.1016/s1074-5521(02)00151-5. URL `https://doi.org/10.1016/s1074-5521(02)00151-5`.

Ping Ge, Shahidul Islam, Cuncong Zhong, and Shaojie Zhang. De novo discovery of structural motifs in rna 3d structures through clustering. *Nucleic acids research*, 46, 03 2018. doi: 10.1093/nar/gky139.

KB Hall. Interaction of rna hairpins with the human u1a n-terminal rna binding domain. *Biochemistry*, 33(33):10076–10088, 1994.

Sven Heinz, Christopher Benner, Nathanael Spann, Eric Bertolino, Yin C Lin, Peter Laslo, Jason X Cheng, Cornelis Murre, Harinder Singh, and Christopher K Glass. Simple combinations of lineage-determining transcription factors prime cis-regulatory elements required for macrophage and b cell identities. *Molecular cell*, 38(4):576–589, 2010.

Laurent Huck, Anne Scherrer, Lionel C Terzi, Arthur E. Johnson, Harris D. Bernstein, Stephen Cusack, Oliver Weichenrieder, and Katharina Strub. Conserved tertiary base pairing ensures proper rna folding and efficient assembly of the signal recognition particle alu domain. *Nucleic acids research*, 32 16:4915–24, 2004.

Arttu Jolma, Jilin Zhang, Estefania Mondragón, Ekaterina Morgunova, Teemu Kivioja, Kaitlin U Laverty, Yimeng Yin, Fangjie Zhu, Gleb Bourenkov, Quaid Morris, et al. Binding specificities of human rna-binding proteins toward structured and linear rna sequences. *Genome research*, 30(7):962–973, 2020.

Ioanna Kalvari, Eric P Nawrocki, Nancy Ontiveros-Palacios, Joanna Argasinska, Kevin Lamkiewicz, Manja Marz, Sam Griffiths-Jones, Claire Toffano-Nioche, Daniel Gautheret, Zasha Weinberg, Elena Rivas, Sean R Eddy, RobertÂ D Finn, Alex Bateman, and Anton I Petrov. Rfam 14: expanded coverage of metagenomic, viral and microrna families. *Nucleic Acids Research*, 49(D1):D192–D200, 11 2020. doi: 10.1093/nar/gkaa1047. URL `https://doi.org/10.1093/nar/gkaa1047`.

Kaitlin U Laverty, Arttu Jolma, Sara E Pour, Hong Zheng, Debashish Ray, Quaid Morris, and TimothyÂ R Hughes. Priesstess: interpretable, high-performing models of the sequence and structure preferences of rna-binding proteins. *Nucleic Acids Research*, 08 2022. doi: 10.1093/nar/gkac694. URL `https://doi.org/10.1093/nar/gkac694`. gkac694.

Sébastien Lemieux and François Major. Automated extraction and classification of rna tertiary structure cyclic motifs. *Nucleic Acids Research*, 34(8):2340–2346, 01 2006. doi: 10.1093/nar/gkl120. URL `https://doi.org/10.1093/nar/gkl120`.

Ronny Lorenz, Stephan H Bernhart, Christian Höner zu Siederdissen, Hakim Tafer, Christoph Flamm, Peter F Stadler, and Ivo L Hofacker. Viennarna package 2.0. *Algorithms for molecular biology*, 6(1):1–14, 2011.

John S. McCaskill. The equilibrium partition function and base pair binding probabilities for rna secondary structure. *Biopolymers*, 29, 1990.

Anton Petrov, Craig Zirbel, and Neocles Leontis. Automated classification of rna 3d motifs and the rna 3d motif atlas. *RNA (New York, N.Y.)*, 19, 08 2013a. doi: 10.1261/rna.039438.113.

Anton I Petrov, Craig L Zirbel, and Neocles B Leontis. Automated classification of rna 3d motifs and the rna 3d motif atlas. *Rna*, 19(10):1327–1340, 2013b.

Mariusz Popenda, Marta Szachniuk, Marek Blazewicz, Szymon Wasik, Edmund Burke, Jacek Blazewicz, and Ryszard Adamiak. Rna frabase 2.0: An advanced web-accessible database with the capacity to search the three-dimensional fragments within rna structures. *BMC bioinformatics*, 11:231, 05 2010. doi: 10.1186/1471-2105-11-231.

Vladimir Reinharz, Antoine Soulé, Eric Westhof, Jérôme Waldispühl, and Alain Denise. Mining for recurrent long-range interactions in rna structures reveals embedded hierarchies in network families. *Nucleic acids research*, 46, 03 2018. doi: 10.1093/nar/gky197.

Elena Rivas, Jody Clements, and Sean R Eddy. A statistical test for conserved rna structure shows lack of evidence for structure in lncrnas. *Nature methods*, 14(1):45–48, 2017.

Roman Sarrazin-Gendron, Hua-Ting Yao, Vladimir Reinharz, Carlos G. Oliver, Yann Ponty, and Jérôme Waldispühl. Stochastic sampling of structural contexts improves

the scalability and accuracy of rna 3d module identification. In Russell Schwartz, editor, *Research in Computational Molecular Biology*, pages 186–201, Cham, 2020. Springer International Publishing.

Bernhard C. Thiel, Roman Ochsenreiter, Veerendra P. Gadekar, Andrea Tanzer, and Ivo L. Hofacker. Rna structure elements conserved between mouse and 59 other vertebrates. *Genes*, 9(8), 2018. doi: 10.3390/genes9080392. URL https://www.mdpi.com/2073-4425/9/8/392.

Ignacio Tinoco and Carlos Bustamante. How rna folds. *Journal of Molecular Biology*, 293 (2):271–281, 1999. doi: https://doi.org/10.1006/jmbi.1999.3001. URL https://www.sciencedirect.com/science/article/pii/S0022283699930012.

Eric Westhof. Isostericity and tautomerism of base pairs in nucleic acids. *FEBS Letters*, 588(15):2464–2469, 2014. doi: https://doi.org/10.1016/j.febslet.2014.06.031. URL https://www.sciencedirect.com/science/article/pii/S0014579314004931. Paris.
